# Indirect cognitive mapping in glioma surgery in patients not eligible for awake craniotomy – how I do it

**DOI:** 10.1007/s00701-025-06706-1

**Published:** 2025-11-07

**Authors:** Patrick Vigren, Hans Lindehammar

**Affiliations:** 1 Department of Neurosurgery, Örebro University Hospital, Örebro, Sweden; 2 Faculty of Medicine, Örebro University, Örebro, Sweden; 3 Department of Neurophysiology, Region Östergötland, Sweden

**Keywords:** Glioma, Glioblastoma, Intraoperative mapping, Cognition

## Abstract

This paper illustrates a method to map cognitive subcortical white matter pathways in brain tumour surgery, in patients not eligible for awake intraoperative mapping. The latter being the gold standard, it is not feasible in all patients. Illustrated by three cases, the presented method includes a preoperative mapping of both motor and subcortical eloquent structures – such as arcuate fasciculus and basal ganglia – subsequentially using subcortical motor mapping as a landmark to indirectly identify the cognitive structures.

**Supplementary Information:**

The online version contains supplementary material available at 10.1007/s00701-025-06706-1.

## Relevant surgical anatomy

**Fig. 1:**
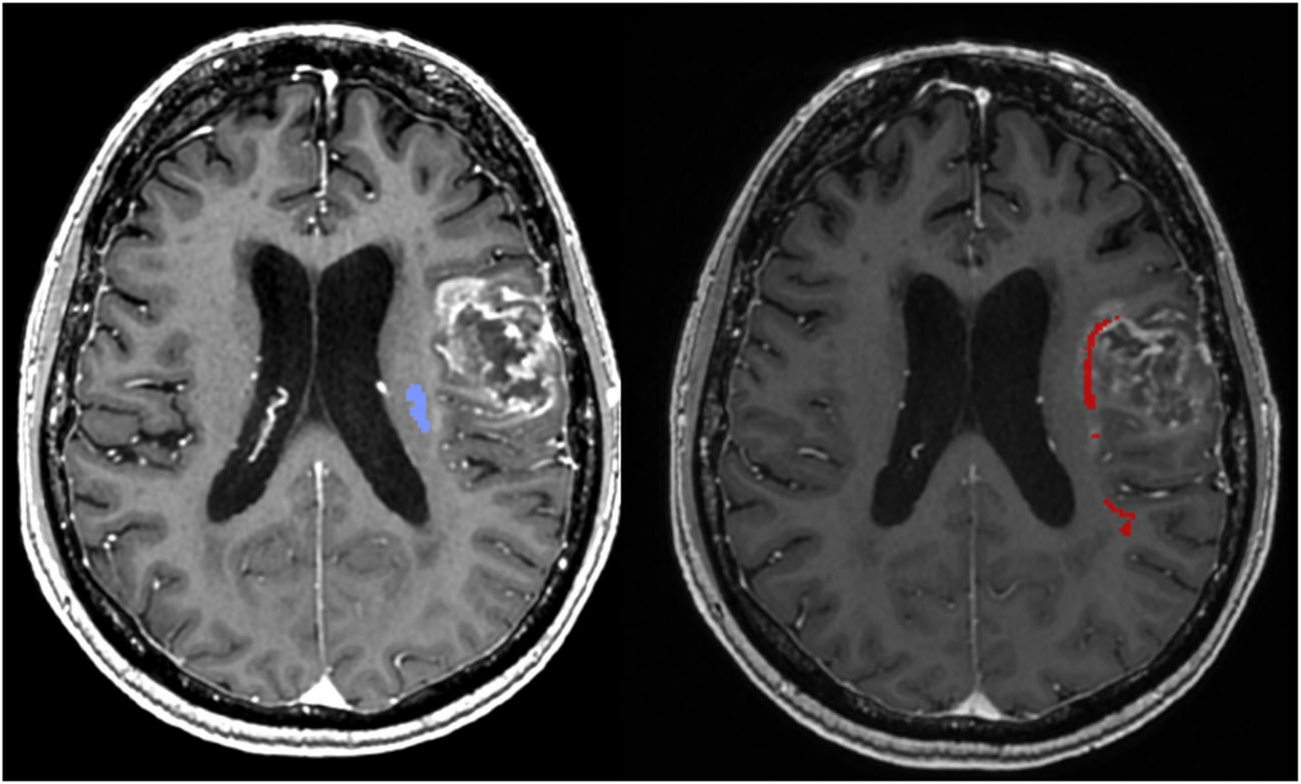
MRI showing a contrast enhancing tumour in the left frontal lobe. Corticospinal tract = purple, arcuate fascicle = red

The basic white matter subcortical pathways of cognitive connectivity in the brain are divided into the ventral stream – including the inferior fronto-occipital fascicle (IFOF), inferior longitudinal fascicle (ILF) and uncinate fascicle (UF) and the dorsal stream – including the arcuate fascicle (FA) and the third superior longitudinal fascicle (SLFIII). [3, 11] Another important pathway is the frontal aslant tract (FAT), not traditionally related to the classical dual streams, however involved in several cognitive functions, but also in higher motor function and related to the supplementary motor area (SMA) syndrome. [1] Classically, the corticospinal tract (CST), with different subcomponents such as the internal capsule, is considered the main motor pathway. [4–6] The anatomical relationships between these pathways are illustrated in Fig. 1 (blue = CST, red = FA), by means of diffusion tensor imaging (DTI), also named fiber tractography.

## Description of the technique

The neurosurgical-anaesthesiological model from our group has previously been described, utilising the asleep-awake-(asleep) model. [9, 10] Although employing the same method regarding neurophysiology and preoperative planning, some patients may not tolerate – or in other ways be ineligible to – awake craniotomy. [2] Thus, the team needs to find alternative ways of protecting important subcortical tracts of cognition and other eloquent structures.

For motor stimulation: using a monopolar subcortical stimulator pen with cathodal stimulation, i.e. stimulating from the cathode to the anode, the subcortical tract can produce an action potential resulting in an EMG signal in corresponding muscles. With the cathodal stimulation, an activation can be seen at different stimulation intensity. To localize the motor pathway (corticospinal tract) within the brain, electrical stimulation delivered by a handheld probe has been used for a long time. The intensity of the current applied can be used to estimate the distance to the motor tract. Electrical stimulation of the motor axons can elicit motor evoked potentials (MEP) recorded from needle electrodes in various target muscles. [7]

Using subcortical electrical stimulation with a monopolar probe and specific stimulation parameters, the relation between stimulation intensity and the distance between the stimulation probe and the motor tract has been established. The stimuli consist of a short train of five pulses with pulse length 500 µs and four msec inter stimulus interval. The stimulating probe is set as the cathode and a distant scalp electrode as the anode. With these parameters there is a current-to-distance relationship, as a rule of thumb, 1 mA correspond to 1 mm. [8]

Anatomically relating the motor pathways/corticospinal tract to the relevant structure for other functions – e.g. arcuate fasciculus, inferior fronto-occipital fascicle, basal ganglia – through preoperative radiology and diffusion tractography imaging will result in a three dimensional functional map. Thus, the motor pathways/corticospinal tract can act as anatomical landmarks to preserve the structure of interest.

In case 1 the patient had a preoperative dysphasia proving the vicinity to and functional relevance of frontal portion of the FA (Fig. 1 = red). Radiologically, there was an oedema affecting the FA. As illustrated in Fig. 1, the distance between the lateral border of the FA (red) and the CST (purple) was 6 mm. Hence, we decided to limit the resection to an activation at 6 mA of the CST. The dysphasia did not deteriorate after the surgery. 95% of the contrast enhancement was removed.

**Fig. 2:**
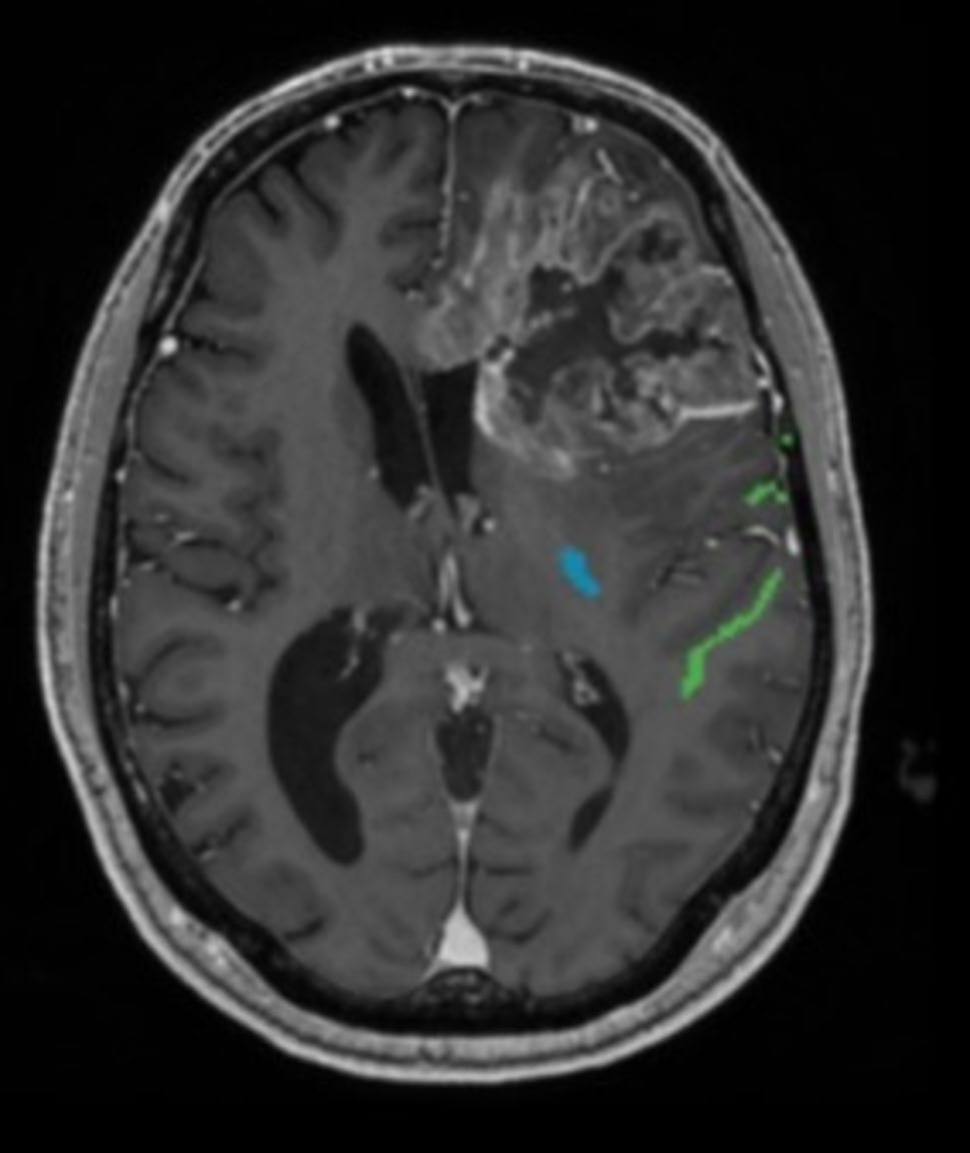
MRI showing a contrast enhancing tumour in the left frontal lobe. Corticospinal tract = blue, arcuate fascicle = green

In case 2, the contrast enhancing part of the tumour was reaching the basal ganglia. The frontal edge of the basal ganglia was at an 12 mm distance from the internal capsule as illustrated in Fig. 2 (blue = CST, green = FA). Thus, we planned to limit the resection to motor activation at 12 mA and 99% of the contrast enhancing tumour could be safely removed. The patient had no new postoperative neurological deficits.

**Fig. 3:**
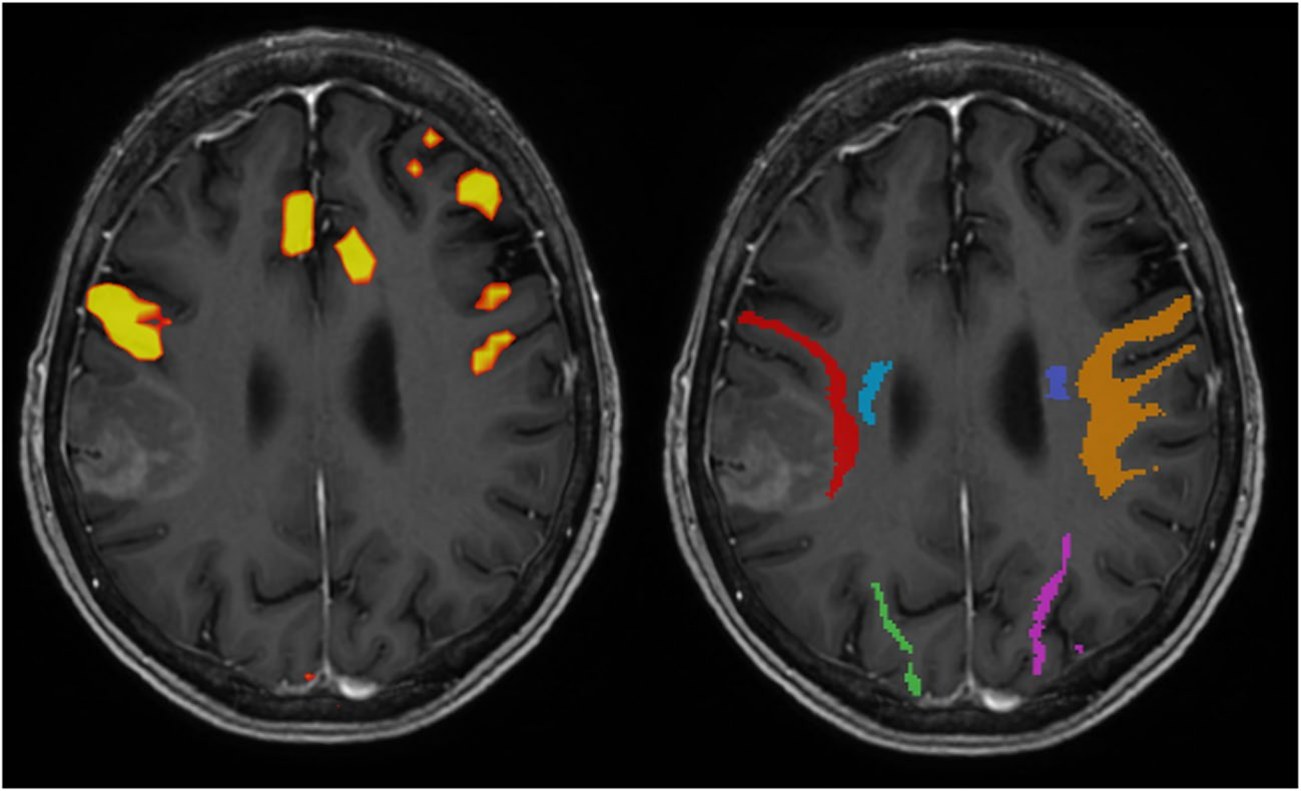
MRI showing a contrast enhancing tumour in the right temporo-parietal lobe. Left: fMRI showing BOLD signal patterns during the word generation task. Right: Right corticospinal tract = blue, right arcuate fascicle = red, right IFOF = green, left corticospinal tract = purple, left arcuate fascicle = orange, left IFOF = magenta

In case 3, a right-handed woman, the tumour was located in the right supramarginal gyrus. The patient did not have preoperative aphasia but the arcuate fascicle was directly adjacent to the medial border of the contrast enhancement (Fig. 3 = left red). Furthermore, fMRI showed strong BOLD signal during word generation frontally to the tumour (Fig. 3 = right). Our conclusion was that the right arcuate fascicle was of great importance to the patient’s speech function. As per department standard, we planned to perform the surgery awake, but the patient had severe preoperative nausea and started vomiting. Thus, we proceeded with the patient intubated. The distance from the lateral limitation of the FA to the lateral limitation of the CST was 10 mm(Fig. 3 = blue). Hence, we stopped the resection when we detected EMG motor activation of the upper extremity at 10 mA. The patient did not show any post-operative dysphasia and postoperative MRI showed total resection of contrast enhancement.

## Indications

Intraoperative mapping in awake patients has been established as the golden standard for preservation of functional structures, especially subcortical white matter tracts, in both low-grade and high-grade gliomas. This is especially true for functions other than motor function, e.g. speech, perception and social cognition. However, in some patients, awake craniotomy is not possible due to a variety of reasons. Some patients might have anaesthesiologically complicating factors, such as a complicated airway or morbid obesity, whilst others might be too cognitively affected to be able to participate in an awake procedure, e.g. not being able to follow instructions.

In the three cases presented in this report, case 1 and 2 were too cognitively affected to participate in an awake procedure. In case 3, an awake procedure was planned with some hesitation from the anaesthesiologist, as the patient had preoperative nausea resistant to antiemetic medication. During the positioning the patient started to vomit, whereas the team unanimously decided to perform the surgery with the patient intubated.

Neuronavigation is most certainly a helpful tool. However, in large tumour resections, brain shift is highly confounding and however accurate the registration, it cannot be trusted at millimetre level after the tumour resection is initiated. Intraoperative MRI can be an additional tool, but the available methods do not include validated tractography protocols and once the resection is continued, the neuronavigation is yet again distorted. Thus, a functional landmark, such as the CST, can be considered a live intracerebral landmark not affected by brain shift.

## Limitations

Certainly, awake mapping is preferred as the patient becomes their own functional template. Considering the individual variation in dependence of specific tracts for specific functions, awake mapping is the only safe – and scientifically strongly supported – method. This is especially true when the correlation between individually relevant function and the tract you are saving is elusive. In case 3, it is possible that the fMRI and DTI did not actually show a relevant tract for the patient’s overall speech function but this was not possible to conclude during surgery with the available information.

## Specific information to the patient

As in all glioma surgery, the patient needs to be thoroughly informed on the expected concomitant treatment effect of the resection and the risk of postoperative functional impairment. However, to our experience of over 300 awake procedures, it is also important to inform the patient of the expected dynamics of postoperative symptoms. As the resection is often very close to the subcortical pathways, transient postoperative deficits are frequently seen. Following postoperative oedema, they not rarely worsen the first 3–4 days and then gradually fully recover. Failing in informing the patient about this might lead to unnecessary discomfort.

## Key points

- Awake intraoperative mapping is the gold standard for cognitive preservation in glioma surgery
- Some patients are not eligible for awake surgery
- Knowledge of subcortical pathways is crucial for presurgical planning
- Preoperative diffusion tensor imaging (DTI) and functional magnetic resonance imaging (fMRI) can create a preliminary functional map
- Presurgical symptoms can imply vicinity to cognitive structures
- Mapping motor pathways can be used as landmark in relation to cognitive structures
- Monopolar subcortical stimulation evaluates distance to motor pathways
- In monopolar subcortical stimulation, activation is approximately 1mA:1mm
- Patients need to be informed about risks of transient and permanent postoperative deficits
- Combining preoperative mapping, neuronavigation and intraoperative motor mapping can be used as a secondary alternative to awake intraoperative mapping

## Supplementary Information

Below is the link to the electronic supplementary material.

Supplementary file1 (MP4 194160 KB)

## Data Availability

No datasets were generated or analysed during the current study.
